# Far from Membranes, BEACH Domains Regulate Stress-Related mRNAs

**DOI:** 10.1371/journal.pbio.1002189

**Published:** 2015-07-02

**Authors:** Richard Robinson

**Affiliations:** Freelance Science Writer, Sherborn, Massachusetts, United States of America

## Abstract

BEACH domain-containing proteins (BDCPs) were thought to be involved in membrane dynamics, but a new study reveals that a plant BDCP helps to regulate transcript stability in response to salt stress, and BDCPs in other organisms may play a similar role.

Sometimes in research, an unexpected finding can not only lead to an advance in one field but also shed light on a mystery in another. That appears to be the case in a new study from Alexandra Steffens, Martin Huelskamp, and colleagues, who show that a protein previously believed to be exclusively involved in membrane dynamics also plays a critical role in regulating salt stress responses in plants. Because this stress response function depends on a domain that is widely distributed and highly conserved throughout eukaryotic life, it seems likely that this domain plays a similar stress-response role elsewhere, explaining previously mysterious consequences of mutations in related proteins in mice.

The protein in question, from the model plant Arabidopsis, is called SPIRRIG and contains the highly conserved BEACH domain. Proteins containing these domains have been known as regulators of various membrane trafficking events, and the authors indeed found a number of membrane-specific binding partners when they performed a binding assay using SPIRRIG's BEACH domain as bait. But they also discovered the domain bound to a nonmembrane protein called decapping protein 1 (DCP1), a core component of P-bodies. They confirmed the result with the entire SPIRRIG protein and showed it was specific for DCP1, not any of several other P-body proteins.

P-bodies are highly dynamic cytoplasmic structures that sequester messenger RNA, thereby temporarily limiting protein synthesis, a common response to various types of stress. To test whether SPIRRIG was involved in stress responses in Arabidopsis, the authors subjected leaves to osmotic stress with either salt or the sugar mannitol. Mannitol had no effect, but salt triggered accumulation of SPIRRIG in P-bodies. Mutations of SPIRRIG resulted in a reduced P-body formation, and mutant plants grew poorly in the presence of salt compared to their wild-type relatives (Fig 1).

**Fig 1:**
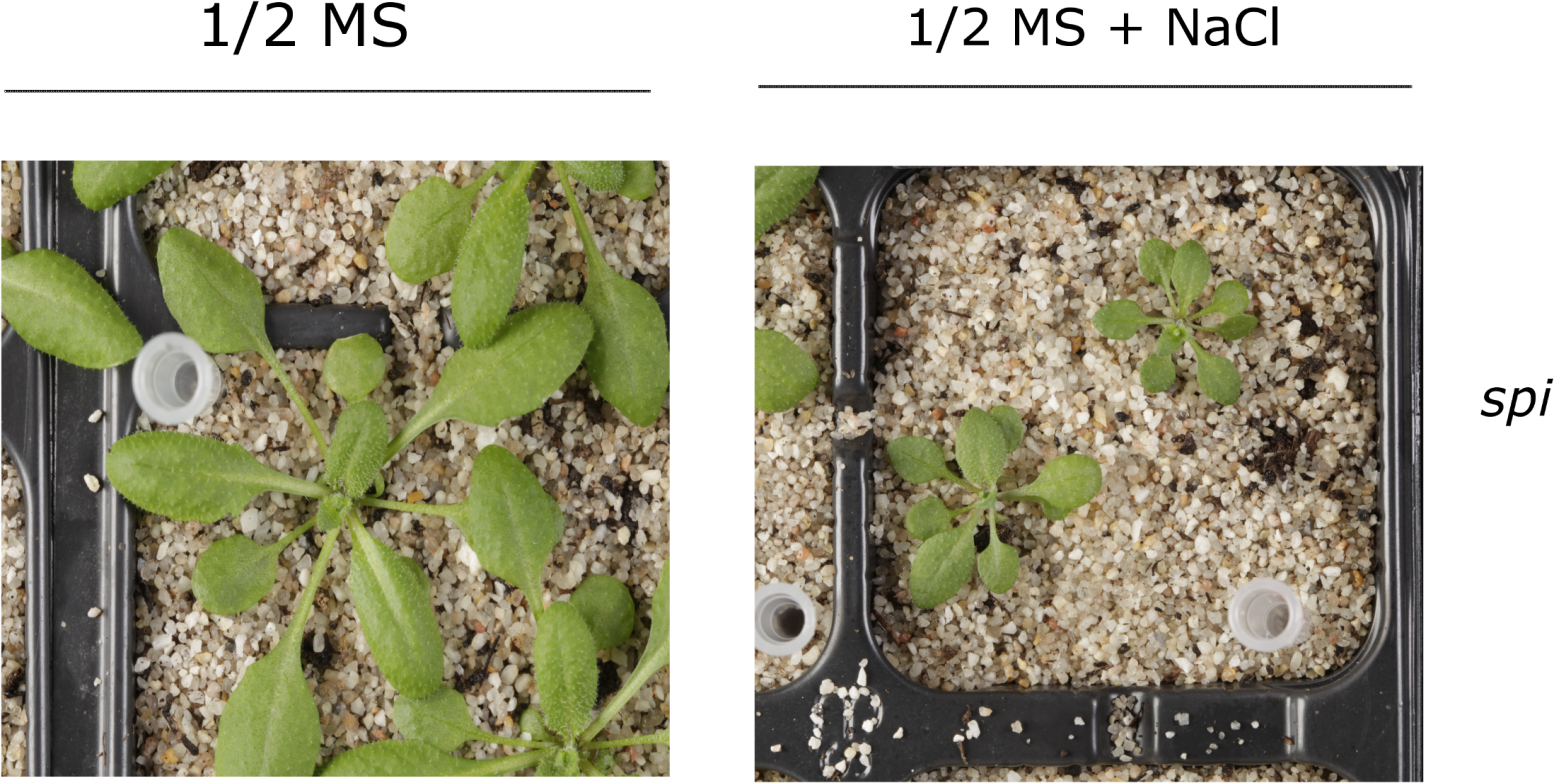
Loss of the SPIRRIG protein leads to salt stress hypersensitivity in *Arabidopsis thaliana*. Presented are soil-grown SPI mutant plants watered with pure nutrient solution (1/2 MS) or nutrient solution supplemented with NaCl (1/2MS + NaCl) to provoke salt stress conditions. *Image Credit: Siegfried Werth.*

At the subcellular level, SPIRRIG mutation in the presence of salt stress affected the expression of multiple genes and the stability of multiple RNA transcripts, many of which, but not all, were directly involved in salt tolerance. Mutation of SPIRRIG reduced sequestration of its mRNA targets into RNA granules and ultimately into P-bodies.

The discovery of a salt stress regulator in Arabidopsis has potential significance in understanding how plants, including agriculturally important ones, are likely to adapt to a world in which changing rainfall patterns will likely increase soil salinity over large areas. But in one final experiment, the authors also found evidence for an even wider significance. They found that SPIRRIG's BEACH domain bound to both mammalian and yeast forms of DCP1, and that a human BEACH-containing protein bound to the Arabidopsis DCP1. Taken together, these results suggest that BEACH domain proteins likely play a similar RNA-sequestering role across the spectrum of eukaryotic organisms, and that understanding the details of this stress response in one organism is likely to shed light on similar responses in many others.
